# One-to-One Embedding between Honeycomb Mesh and Petersen-Torus Networks

**DOI:** 10.3390/s110201959

**Published:** 2011-02-01

**Authors:** Jung-Hyun Seo, Hyun Sim, Dae-Heon Park, Jang-Woo Park, Yang-Sun Lee

**Affiliations:** 1 Department of Computer Engineering, National University of Sunchon, Maegok 315, Sunchon, Jeonnam, Korea; E-Mails: jhseo@scnu.ac.kr (J.-H.S.); simhyun@scnu.ac.kr (H.S.); 2 Department of Information and Communication Engineering, National University of Sunchon, Maegok 315, Sunchon, Jeonnam, Korea; E-Mails: dhpark@scnu.ac.kr (D.-H.P.); jwpark@scnu.ac.kr (J.-W.P.); 3 Department of Information and Communication Engineering, Chosun Universtiy, 375 Seosuk-Dong, Dong-Gu, Gwangju, Korea

**Keywords:** embedding, honeycomb mesh, Petersen-Torus, interconnection network parallel processing

## Abstract

As wireless mobile telecommunication bases organize their structure using a honeycomb-mesh algorithm, there are many studies about parallel processing algorithms like the honeycomb mesh in Wireless Sensor Networks. This paper aims to study the Peterson-Torus graph algorithm in regard to the continuity with honeycomb-mesh algorithm in order to apply the algorithm to sensor networks. Once a new interconnection network is designed, parallel algorithms are developed with huge research costs to use such networks. If the old network is embedded in a newly designed network, a developed algorithm in the old network is reusable in a newly designed network. Petersen-Torus has been designed recently, and the honeycomb mesh has already been designed as a well-known interconnection network. In this paper, we propose a one-to-one embedding algorithm for the honeycomb mesh (HMn) in the Petersen-Torus PT(n,n), and prove that dilation of the algorithm is 5, congestion is 2, and expansion is 5/3. The proposed one-to-one embedding is applied so that processor throughput can be minimized when the honeycomb mesh algorithm runs in the Petersen-Torus.

## 1. Introduction

Computers are widely used in our everyday life. Almost all computers have one processor that is able to perform instructions sequentially. There soon will be a time when sequential computers reach their physical limits in CPU speed and memory space. One alternative is parallel processing using several processors. The role of interconnection network, which displays the interconnection structure of processors composing the large-scale parallel processing system in a graph, is very important so that the system can efficiently accept various application algorithms in engineering and scientific fields and fully exercise its performance. Interconnection network consists of a set of processors, local memory, and a communication link between processors for data transfer. An interconnection network is divided into dynamic interconnection network and static interconnection network. Static interconnection networks cannot be connected with other processors again because the communication link between two processors is manual, while in a dynamic interconnection network, communication links can be linked with other processors by means of SE (Switching Elements). Dynamic interconnection networks can be classified into single stage, multi stage, cross bar, and cellular array. The single stage contains recirculating shuffle-exchange interconnection network, and the multi stage contains data manipulator, flip, indirect binary n-cube, omega, clos, and cantor. Cellular array includes near-neighbor connection [1]. According to the composition of nodes and edges composing the network, static interconnection networks can be classified into mesh class, hypercube class, and star graph class. Mesh classes include torus, honeycomb mesh [2], diagonal mesh [3], hexagonal mesh [4], Petersen-Torus [5] *etc.*, hypercube class includes hypercube [6], folded hypercube [7], multiply-twisted-cube [8], recursive circulant [9], Cayley Graph of Degree Three [10], *etc.*, and star graph class includes star graph [11], macro-star [12], transposition graph [13], matrix-star graph [14], *etc.*

In a mesh structure, extension of networks is easy since the degree is constant, and the structure, which is highly available in the VLSI circuit design, has been broadly used and commercialized until now. Since a low dimension mesh can be readily designed and is very useful in terms of algorithm, it is frequently used as interconnection network for parallel computers. The higher the dimensions of the mesh are, the shorter is diameter and the larger is bisection width and several parallel algorithms can be rapidly performed, but it requires a huge cost [15]. Honeycomb mesh is proposed as an interconnection network, which is characterized by more economic network cost, better location of mobile base station, and application fields such as computer graphic, image processing *etc.* An interconnection network can be modeled as an undirected graph G = (V,E). Each processor Pi is an element of a node set V, and two processors Pi and Pj are connected by communication link (Pi, Pj). The number of edges incident to the node Pi is defined as degree of the node.

Embedding means the logical matching of two interconnection networks. For nodes and edges composing the interconnection networks, the algorithm developed in G is reusable in the algorithm H by matching the node of G with that of H and the edge of G with the path of H. Given a guest graph G and a host graph H, embedding of G in H is described by an ordered pair (Φ, Ψ), where Φ maps each node of G to a node of H and Ψ maps each edge (u, v) of G to a path of H from nodes Φ(u) to Φ(v) (hereafter referred to as Ψ-path). Dilation of the edge (u, v) is the length of the Ψ-path in H, and dilation of the embedding (Φ, Ψ) is the largest value among dilations for all edges of G. Congestion of the edge of H is Ψ-paths traversing an edge of H, and congestion of the embedding (Φ, Ψ) is the largest value among congestions for all edges of H. Expansion of the embedding (Φ, Ψ) is a ratio of the number of nodes of G to that of H [16,17]. The measures to evaluate the embedding algorithm are dilation, congestion, and expansion. The closer the values are to 1, the better the embedding algorithm is.

If expansion is less than 1, it is impossible to efficiently apply the algorithm designed at *G* to *H*. If expansion is more than 1, non-efficiency occurs that is, all nodes of H are not available when the algorithm designed at *G* is executed at *H*. It is recommended that expansion be exactly 1. The minimum value of dilation is 1, and the higher is dilation, the longer is message transmission time as much as dilation when the algorithm designed at *G* is applied to H. The optimal value of congestion is 1, and the higher is dilation, the more is transmission traffic. Optimal congestion is 1, and the higher is congestion, the more the transfer traffic is. The higher the dilation is, the longer the transfer time in store-and-forward routing is, therefore once the mode of wormhole routing is suitable and congestion is high, the possibility of message dead-lock increases in wormhole routing. Message transfer in multi-computing system is divided into circuit switching and packet switching. The former makes two processors exclusively available while transferring message by setting circuits toward the destination. The latter is classified into store-and-forward routing, virtual cut-through routing, and wormhole routing. Store-and-forward routing has longer message delay and requires many memory storage devices, which is designed to save message in the message storage device of the middle node on the path when the packet is transferred and transfer it again. Wormhole routing is designed to divide a packet into small units called flit to be given support from the router, when the header flit just in front of the message decides routing and the other flits successively follow the header flit. In this study, dilation and congestion were embedded at 5 or less to satisfy both the routing system.

Studies on embedding between interconnection networks include embedding tree, cycle, mesh, hypercube, and star graph into other interconnection networks [17–20], and embedding among mesh, hypercube, and star graph [21,22]. In a study on embedding among mesh classes, the embedding measures are mostly one place integers. In [23], a (*n* − 1) × *n* mesh was embedded in a *n* × (*n* − 1) mesh at *expansion* 1 and *dilation* 2, and *j* × *k* mesh in *n* × *n* mesh at *expansion* 1 and *dilation* 3(*j* × *k* = *n* × *n*). In [24], two-dimensional *h* × *w* mesh was embedded in *h* × ‘*w*’ mesh at *dilation* 2(*hw* ≤ *h*‘*w*’, *w*’ < *w*). In [25], a (5m,2n) Torus is embedded in Petersen-Torus PT(m,n) with dilation 5, congestion 5, and expansion 1. In [26], *k*-dimensional Torus *G* was embedded in *H* at *dilation* 1 and *congestion* 1(If the number of nodes of G is equivalent to or more than that of H). In [27], (3*n*,2*n*) Torus was embedded in n-dimensional hexagonal honeycomb Torus at *dilation* 2, *congestion* 4, *expansion* 1. In [28], two-dimensional *h* × *w* mesh was embedded one-to-one in *s*×*s* mesh at *dilation* 6(*h* × *w > s* × *s*).

This paper is composed as follows. Section 2 introduces the Petersen-Torus and the honeycomb mesh network. Section 3 proposes an algorithm of embedding the honeycomb mesh in the Petersen-Torus network, and finally, conclusions are given.

## 2. Related Work

### 2.1. Petersen-Torus

The Petersen graph is the graph with the most desirable network cost to the number of nodes, such that it has the most number of nodes (10) among graphs having degree 3 and diameter 2. Taking advantage of the Petersen graph, PT was designed in place of the Petersen graph having 10 nodes per node of torus. A Peter-Torus network has a smaller diameter and a smaller network cost than a honeycomb torus with same node number [5].

**Figure 1.**
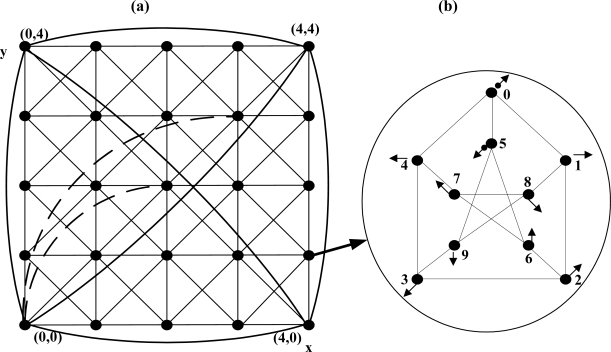
Petersen-Torus PT(5,5): **(a)** PT(5,5), **(b)** Petersen Graph.

The Petersen-Torus PT(m,n) (m,n ≥ 2) sets the Petersen graph [Figure 1(b)] as a basic module, arranges m(x axis) × n(y axis) modules on grid points, and connects them under an edge definition. In this paper, PT(m,n) is described by mapping in a two-dimensional graph as shown in Figure 1(a). A unit Petersen graph is set as module, and module is located on the intersecting point of x and y. The address of module is indicated as (x,y) and the node address as (x,y,p). x is the coordinates of x axis of module and y is the coordinates of y axis of module, p is the node address in Petersen graph. The Petersen-Torus network is defined as PT(m,n) = (Vpt, Ept). The node definition of PT(m,n) is:

Vpt={(x,y,p),0≤x<m,0≤y<n,0≤p≤9}

The edges of PT(m,n) are divided into internal edges and external edges. The edges connecting the nodes belonging to the same basic module are called internal edges, in which the edges of the Petersen graph are used as they are. The edges connecting the nodes belonging to other basic modules are called external edges. Edges are defined in the following. The symbol ‘%’ is the remainder operator in the following equations. (1) The longitudinal edge is ((x,y,6), (x,(y + 1)%n,9)). (2) The latitudinal edge is ((x,y,1), ((x + 1) %m,y,4)). (3) The diagonal edge is ((x,y,2), ((x + 1)%m,(y + 1)%n,3)). (4) The reverse diagonal edge is ((x,y,7), ((x – 1 + m)%m,(y + 1)%n,8)). (5) The diameter edges is ((x,y,0), ((x + ⌊*m*/2⌋)%m,(y + ⌊*n*/2⌋)%n,5)).

Figure 1(a) expresses modules in grid points in Petersen-Torus *PT*(5,5). For all modules except verge, edges except diameter edge are drawn, and only the diameter edges of the module (0,0) are drawn in thick dashed lines (short lines are repeated regularly). Wraparound edges are omitted in the modules of the edges but several wraparound edges are drawn in thick solid lines in the modules on the four vertexes. Seen from a Petersen graph [Figure 1(b)] of the module of Petersen-Torus *PT(m,n)*, Nodes 1, 4 are latitudinal edges, nodes 6, 9 longitudinal edges, nodes 2, 3 diagonal edges, nodes 7, 8 reverse diagonal edges, and nodes 0, 5 are incident diameter edges. *PT(m,n)* is a regular graph where the number of nodes is 10*mn*, the number of edges 20*mn*, connectivity 4, and degree 4.

Routing between two nodes in the same module is called internal routing, while routing between two nodes in the different modules is called external routing. The basic strategy of routing is to, when routing to modules each distant by 1 on x axis and y axis, use diagonal edge instead of latitudinal edge and longitudinal edge. Internal routing is in [5].

Let *U*(*x_1_, y_1_, p_1_*) is source node, *V*(*x_2_, y_2_, p_2_*) is destination node, and *T*(*x′, y′, p′*) is intermediate node. *dx* = *(x_2_ − x_1_* + *m*) / *m and dy* = (*y_2_ − y_1_*+*n*) / *n. dm* = min(*dx,dy*) and *dr* = *dx* − *dy*. *dx* is x axis distance in a direction that *x* coordinates increase. *dy* is *y* axis distance in a direction that *y* coordinates increase. The basic strategy of routing is to, when routing to modules each distant by 1 on x axis and y axis, use diagonal edge instead of latitudinal edge and longitudinal edge. Routing algorithm is summarized as below:

**Table:** 

[Step 1]	Internal routing in source module
	Routing from source node to intermediate node incident to diagonal or reverse diagonal or latitudinal or longitudinal edge
[Step 2]	External routing with diagonal edge or reverse diagonal edge
	Routing with diagonal or reverse diagonal edge in *dm*
[Step 3]	External routing with latitudinal edge or longitudinal edge
	Routing in *dm*-*dx* with latitudinal edge or Routing in *dm*-*dy* with latitudinal edge
[Step 4]	Internal routing in destination module
	Routing to destination node from intermediate node connected to diagonal or reverse diagonal or latitudinal or longitudinal edge

### 2.2. Honeycomb Mesh

**Figure 2.**
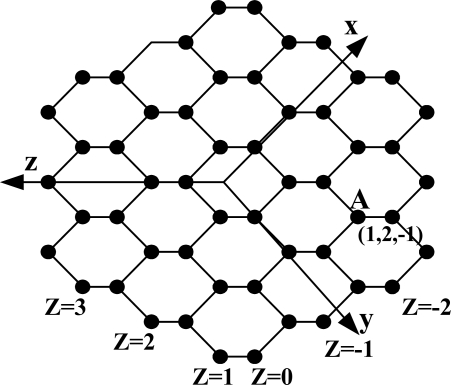
Honeycomb Mesh *HM*_3_.

HMn is made by the following method. HM1 is in form of a hexagon. HM2 is made by attaching each hexagon to the outside of the six edges of HM1. HM3 is made by attaching each hexagon to the outside of the edge of HM2. In the same way, HMn is made by attaching each hexagon to the outside of the edge of HMn-1. Figure 2 shows HM3. A honeycomb mesh [2] can be made in three ways by using a hexagon and according to these ways, is divided into three classes: Honeycomb Hexagonal Mesh (HHM), Honeycomb Rhombic Mesh (HRoM), and Honeycomb Rectangular Mesh (HReM). Each has degree 3, and once wraparound edge is added, it changes into honeycomb Torus. HHM is simply called honeycomb mesh (*HM*). The honeycomb mesh is a bipartite graph. All nodes can be subdivided into two groups, which will be called black and white nodes, such that any edge joins a black and a white node. Vertex and edge symmetric honeycomb torus is obtained by adding wraparound edges to the honeycomb mesh.

The honeycomb mesh *HM_n_* consists of 6*n*^2^ nodes and 9*n*^2^ − 3*n* edges and is indicated in *HM_n_* = (*V_hm_*,*E_hm_*). A node set *V_hm_* and an edge set *E_hm_* are defined as follows:

$$V_{\mathrm{hm}}=\{(u,v,w)|(-n+1\leq u,v,w\leq n,1\leq u+v+w\leq 2)\}$$

$$E_{\mathrm{hm}}=\{((u,v,w),(u^{\prime },v^{\prime },w^{\prime }))||u-u^{\prime }|+|v-v^{\prime }|+|w-w^{\prime }|\;=1\}$$

The node address of HMn is indicated in (u,v,w). Address assignment method of HM is as follows: As seen from Figure 2, the crossing of the x, y, and z axes is deemed as start of each axis. Node address is indicated in (u,v,w), u value of the node which is first met in the x axis from the start point is 1, and u = u + 1 at the address value of the previous node per node which is met in movement. In a reverse direction, u value of the node which is first met in movement is 0, and u = u − 1 at the address value of the previous node per node which is met in movement. In the y(v) axis and the z(w) axis, the address is assigned in the same manner as the x axis. Node A is indicated as an example in Figure 2. For all nodes placed in a zigzag form which meet with the z axis at a right angle, the address value of w is same. When u + v + w = 1, nodes adjacent to the node (u,v,w) are (u + 1,v,w), (u,v + 1,w), and (u,v,w + 1), and when u + v + w=2, nodes adjacent to the node (u,v,w) are (u − 1,v,w), (u,v − 1,w), and (u,v,w − 1).

Let (u, v, w) be the source node, (u′, v′, w′) is the destination node. *du* = u′ − u, *dv* = v′ − v and *dw*=w′ − w. the shortest path between the two nodes consists of |*du*| edges parallel to x-axis, |*dv*| edges parallel to y–axis and |*dw*| edges parallel to z-axis. The routing algorithm checks at each current node which of the edge directions x, y, or z(in this order) would reduce the distance to the destination, and will send the message on that edge. At least one of the edge directions would lead to a node closer to the destination [2].

## 3. Embedding HM (Honeycomb Mesh) in PT (Petersen-Torus)

**Figure 3.**
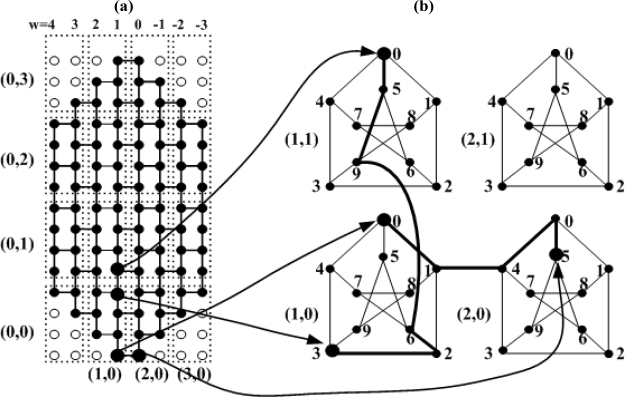
Embedding Honeycomb Mesh *HM*_4_ in Petersen-Torus. **(a)** *HM*_4_ **(b)** *PT* (4,4).

The basic strategy to embed HM in PT is mapping the set of HM (hereafter referred to as a BMH) to the basic module of PT (hereafter referred to as a BMP). In Figure 2, nodes on the zigzag line being at a right angle to the *z*-axis are spread to be on the same vertical line, which is as shown in Figure 3(a). For simplicity of the embedding algorithm, HM as shown in Figure 2 is indicated again in Figure 3(a), and the embedding algorithm shown in Figure 3(a) is embedded in PT. Figure 3(a) shows how BMH is divided in HM. In Figure 3(a), the square shown in dotted line, which is composed of 8 nodes, is BMH, and the dotted line is not an edge but just an indication for division.

**Lemma 1.** A BMH is embedded in a BMP with dilation of 2, congestion of 2 and expansion of 5/4.

**Figure 4.**
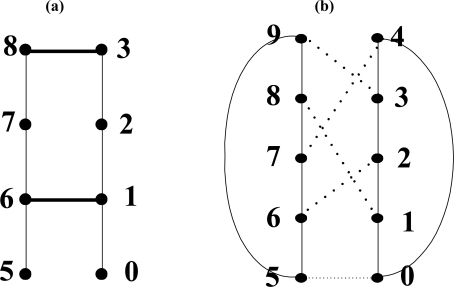
BMH and BMP **(a)** BMH **(b)** BMP.

*Proof*. Figure 4 shows a BMH and a BMP. Nodes of the BMH are one-to-one mapped to nodes of the BMP having the same address. The number of nodes of the BMH is 8 and that of the BMP is 10. Therefore expansion is 5/4.

Assuming that node address of the BMH is a, length of the paths of the BMP in which the (a, a + 1) edges of the BMH are mapped is 1. Dilation and congestion of the (a, a + 1) edges are each 1. Except the (a, a + 1) edges, dilation and congestion of the two edges (1,6) and (3,8) are as described in the following. The edge (1,6) of the BMH is mapped in the path ((1,2), (2,6)) of the BMP with dilation of 2. The edge (3,8) of a BMH is mapped in the path ((3,9), (9,8)) of the BMP with dilation of 2. Therefore the BMH is embedded in the BMP with dilation of 2. Congestion is 2 at the edges (1,2) and (8,9) of the BMP. Therefore the BMH is embedded in the BMP with congestion of 2.

**Theorem 1.** *HM_n_* is embedded in *PT*(*n,n*) with dilation of 5, congestion of 2, and expansion of 5/3 (*n* is an even number).

*Proof.* The number of nodes for *HM_n_* is 6*n*^2^ and that for *PT*(*n,n*) is 10*n*^2^. Therefore expansion is 5/3. In a honeycomb mesh, nodes having the same *w*(=z) stand vertically, as seen from Figure 3(a). The address of the HM node is indicated by (*u,v,w*). The number of nodes at each row is 4*n* − 1 in Figure 3(a). The black node is the HM node, while the white node is the virtual node. *Bu* = −*n* + 1 − ⌊*w*/2⌋, *bv* = *n* − ⌊*w*/2⌋, *k* = *n* − *w*. The address of all nodes positioned at the bottom is (*bu*,*bv*,*w*) (−*n* + 1 ≤ *w* ≤ *n*). In Figure 3(a), when *w* is 1, the address of the node positioned at the bottom is (−3,4,1), and when *w* is 0, the address of the node positioned at the bottom is (−3,4,0). When *k* is an odd number, the node address which goes through *v* = *v* − 1 at the address of the bottom node in an upward direction becomes the next node address, and node address which goes through *u* = *u* + 1 at the node address becomes the next node address. Address of the next node is obtained by repeating this procedure. When *k* is an even number, node address which goes through *u* = *u* + 1 at the address of the bottom node in an upward direction becomes the next node address, and node address which goes through *v* = *v* − 1 at the node address becomes the next address. Address of the next node is obtained by repeating this procedure, as well.

Node (*u,v,w*) of HM is mapped into node (*x,y,p*) of PT as follows: *x* = ⌊*k*/2⌋. When *k* is an odd number, *y* = ⌊*u* − *bu*/2⌋, and when *k* is an even number, *y* = ⌊*bv* − *v*/2⌋. In Figure 3, the address indicated in (number, number) is that of PT basic module into which HM basic module is to be mapped. How PT basic module maps 8 nodes in HM basic module is as described below. In the following equations, ‘%’ is modular operator.

When *k* is an odd number:

Under (*u* − *bu*) % 2 = 0, if (*bv* − *v*) % 2 = 0, *p* = 0, and if (*bv* − *v*)%/ 2 = 1, *p* = 1.
Under (*u* − *bu*) /%2 = 1, if (*bv* − *v*) /%2 = 1, *p* = 2, and if *(bv* − *v*)%/ 2 = 0, *p* = 3.

When *k* is an even number,

Under (*bv* − *v*) /%2 = 0, if (*u* − *bu*)%/ 2 = 0, *p* = 5, and if (*u* − *bu*)%/ 2 = 1, *p* = 6.
Under (*bv* − *v*) /%2 = 1, if (*u* − *bu*) % 2 = 1, *p* = 7, and if (*u* − *bu*) % 2 = 0, *p* = 8.

This method is mapping of the embedding method shown in Figure 3 by using the HM address. As mapping of HM basic module into PT basic module has already been demonstrated in Lemma 1, mapping of edge between HM basic modules is described. The edge between HM basic modules is divided into four: First is HM edge [(*u,v,w*), (*u,v,w* − 1)] if three conditions are met (*k* is an odd number, (*u* − *bu*)/2 = 0, and *(bv* − *v*)/2 = 0). Second is HM edge [(*u,v,w*), (*u,v,w* − 1)] if three conditions are met (*k* is an odd number, (*u* − *bu*)/2 = 1, and (*bv* − *v*)/2 = 1). Third is HM edge [(*u,v,w*), (*u* + 1,*v,w*)] if three conditions are met [*k* is an odd number, (*u* − *bu*)/2 = 1, and (*bv* − *v*)/2 = 0]. Fourth is HM edge [(*u,v,w*), and (*u,v* − 1,*w*)] if three conditions are met [*k* is an even number, (*u* − *bu*)/2 = 0, (*bv* − *v*)/2 = 1]. The former two edges are between the HM basic module mapped into the PT basic module and the HM basic module mapped into the PT basic module (2,0) as shown in Figure 3(a), while the latter two edges are between the basic module mapped into the PT basic module (1,0) and the HM basic module mapped into the PT basic module (1,1).

**Case 1.** If three conditions are met [*k* is an odd number, (*u* − *bu*)/2 = 0, (*bv* − *v*)/2 = 0].

The case meeting the above three conditions is the edge [(*u,v,w*), (*u,v,w* − 1)] between the HM basic module and the adjacent right basic module as shown in Figure 3 (a). If *k* is an odd number, assuming that HM node (*u,v,w*) is mapped into PT basic module (*x,y*), HM node (*u,v,w* − 1) is mapped into PT basic module (*x* + 1,*y*). If *k* is an odd number, (*u* − *bu*)/2 = 0, and (*bv* − *v*)/2 = 0, node (*u,v,w*) is mapped into PT node (*x,y*,0), and node (*u,v,w* − 1) is mapped into PT node (*x +* 1*,y*,5). The path of PT mapped into the HM edge [(*u,v,w*), (*u,v,w* − 1)] are (*x,y*,0), (*x,y*,1), (*x* + 1,*y*,4), (*x* + 1,*y*,0), and (*x* + 1,*y*,5), and length of the path is 4.

**Case 2.** If three conditions are met (*k* is an odd number, (u − bu)/2 = 1, and (bv − v)/2 = 1).

The case meeting the above three conditions is the edge between the HM basic module and the adjacent right basic module as shown in Figure 3(a). If *k* is an odd number, assuming that HM node (*u,v,w*) is mapped into PT basic module (*x,y*), HM node (*u,v,w* − 1) is mapped into PT basic module (*x* + 1,*y*). If *k* is an odd number,, (u − bu)/2 = 1, and (bv − v)/2 = 1, node (*u,v,w*) is mapped into PT node (*x,y*,2), and node (*u,v,w* − 1) is mapped into PT node (*x +* 1*,y*,7). The path of PT mapped into the HM edge [(*u,v,w*), (*u,v,w* − 1)] are (*x,y*,2), (*x,y*,1), (*x* + 1,*y*,4), and (*x* + 1,*y*,7), and length of the path is 3.

**Case 3.** If three conditions are met [*k* is an odd number, (u − bu)/2 = 1, and (bv − v)/2 = 0].

The case meeting the above three conditions is the edge [(*u,v,w*), (*u* + 1,*v,w*)] between the HM basic module and the adjacent right basic module as shown in Figure 3(a). If *k* is an odd number, assuming that HM node (*u,v,w*) is mapped into PT basic module (*x,y*), HM node (*u* + 1,*v,w*) is mapped into PT basic module (*x,y* + 1). If *k* is an odd number, (u − bu)/2 = 1, and (bv − v)/2 = 0, node (*u,v,w*) is mapped into PT node (*x,y*,3), and node (*u* + 1,*v,w*) is mapped into PT node (*x,y* + 1,0). The path of PT mapped onto the HM edge [(*u,v,w*), (*u* + 1,*v,w*)] are (*x,y*,3), (*x,y*,2), (*x,y*,6), (*x,y* + 1,9), (*x,y* + 1,5), and (*x,y* + 1,0), and length of the path is 5.

**Case 4.** If three conditions are met [*k* is an even number, (u − bu)/2 = 0, and (bv − v)/2 = 1].

The case meeting the above three conditions is the edge [(*u,v,w*), (*u,v* − 1,*w*)] between the HM basic module and the adjacent right basic module as shown in Figure 3 (a). If *k* is an even number, assuming that HM node (*u,v,w*) is mapped into PT basic module (*x,y*), HM node (*u,v* − 1,*w*) is mapped into PT basic module (*x,y* + 1). If *k* is an even number, (u − bu)/2 = 0, and (bv − v)/2 = 1, node (*u,v,w*) is mapped into PT node (*x,y*,8), and node (*u,v* − 1,*w*) is mapped into PT node (*x,y* + 1,5). The path of PT mapped into the HM edge ((*u,v,w*), (*u,v* − 1*,w*)) are (*x,y*,8), (*x,y*,7), (*x,y*,6), (*x,y* + 1,9), and (*x,y* + 1,5), and length of the path is 4.

As seen from Case 1 and Case 2, HM edge ((*u,v,w*), (*u,v,w* − 1)) is embeddable into PT at dilation 4, and under Case 3, HM edge ((*u,v,w*), (*u* + 1,*v,w*)) is embeddable into PT at dilation 5, and under Case 4, HM edge ((*u,v,w*), (*u,v* − 1,*w*)) is embeddable into PT at dilation 4. As described in Lemma 1, HM basic module is embeddable into PT basic module at dilation 2 thus HM is embeddable into PT at dilation 5.

Under Lemma 1, HM basic module is embeddable into PT basic module at congestion 2, and the path of PT for the two edges corresponding to Case 1 and Case 2 include the edge ((*x,y*,1), (*x* + 1,*y*,4)), and the path of PT for the two edges corresponding to Case 3 and Case 4 include the edge ((*x,y*,6), (*x,y* + 1,9)), thus HM is embeddable into PT at congestion 2.

**Corollary 1** Honeycomb mesh *HM_n_* embedded in *PT*(*n,n*) at 3 or less of average dilation.

*Proof*. In all HM basic modules, dilation into PT is same, therefore one basic module average is the whole average. As demonstrated in Lemma 1, 6 edges in HM basic module are embedded into PT at dilation 1 and two edges into PT at dilation 2. As demonstrated in Theorem 1, one edge between HM basic modules is embedded into PT at dilation 3, one edge into PT at dilation 5, and two edges at dilation 4. Thus embedding at 3 or less of average dilation is possible.

For example, as shown in Figure 3, HM node (−3,4,1) is mapped into PT node (1,0,0), and HM node (−3,4,0) into PT node (1,1,5). The path of PT mapped into the HM edge ((−3,4,1), (−3,4,0)) are (1,0,0), (1,0,1), (1,1,4), (1,1,0), and (1,1,5), and length of the path is 4. The HM node (−2,3,1) is mapped into the PT node (1,0,2), and the HM node (−2,3,0) into the PT node (1,1,7). The path of PT mapped into the HM edge [(−2,3,1), (−2,3,0)] are (1,0,2), (1,0,1), (1,1,4), and (1,1,7), and length of the path is 3. All the path of PT into which the two HM edges are mapped pass through the edge [(1,0,1), (1,1,4)].

## 4. Comparative Analysis with Other Interconnection Networks

**Figure 5.**
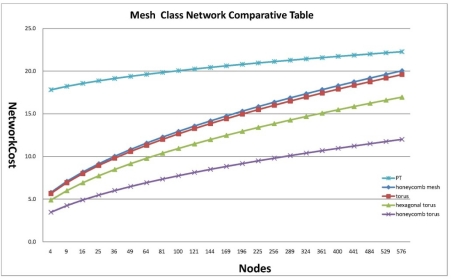
Mesh Class Network Comparative Table.

Network cost is indicated by a multiple of diameter and degree. Diameter indicates a maximum distance of the shortest route linking two nodes, which can be an effective reference to measure message passing as a lower limit of latency required to disseminate information in the whole interconnection network, and degree is the number of pins composing the processor when a parallel computer is designed with a given interconnection network as a factor to determine the complexity of routing control logic, which is a reference to measure the cost of hardware used to implement an interconnection network. Therefore network cost is the most critical factor to measure an interconnection network. To demonstrate that Petersen-Torus suggested in this paper based on the results of previous studies is suitable for implementation of a large-scale system for parallel processing, it is proven to be superior to the previously proposed mesh classes of honeycomb mesh, tours, hexagonal tours and honeycomb tours in terms of network cost as mentioned in Table 1. For analysis of network cost for an interconnection network, cases of the same number of nodes are compared in Figure 5.

## 5. Conclusion

Embedding between two networks is a meaningful job to make a designed parallel algorithm reusable. The proposed embedding algorithm can be available in both a wormhole routing system and a store-and-forward routing system by embedding the generally known honeycomb mesh network in Petersen-Torus with dilation and congestion of 5 or less. Also, the processor throughput could be minimized through one-to-one embedding. Further studies on embedding from Petersen-Torus in other interconnection networks are required to be made so that the algorithms developed in Petersen-Torus can be reusable in another interconnection network. As a result shown above, Peterson-Torus network can be applied to Wireless Sensor Network and it is expected to provide better performance compared to honeycomb mesh algorithm. Our future research will conduct Sensor Network routing with the Peterson-Torus algorithm and will show simulation results for the test of the performance.

## Figures and Tables

**Table 1.** Other Interconnection Network costs *vs*. Petersen-Torus.

	Degree_i_	Diameter	Network Cost
*Honeycomb mesh*	*3*	$1.63\sqrt{N}$	$4.90\sqrt{N}$
*Torus*	*4*	$\sqrt{N}$	$4\sqrt{N}$
*Hexagonal torus*	*6*	$0.58\sqrt{N}$	$3.46\sqrt{N}$
*Honeycomb tours*	3	$0.81\sqrt{N}$	$2.45\sqrt{N}$
*PT*	4	$0.32\sqrt{N}+4$	$1.28\sqrt{N}+16$
